# Treacher Collins syndrome – a case report

**DOI:** 10.1515/crpm-2020-0009

**Published:** 2022-12-19

**Authors:** Magda Fraszczyk-Tousty, Agata Jankowska, Joanna Tousty, Piotr Tousty, Beata Łoniewska

**Affiliations:** Department of Neonatal Diseases, Pomeranian Medical University, Szczecin, Poland; Department of Gynecology and Obstetrics, Pomeranian Medical University, Szczecin, Poland

**Keywords:** Francescchetti-Zwahlen-Klein syndrome, mandibulofacial dysostosis, Treacher-Collins syndrome

## Abstract

**Objectives:**

Treacher Collins syndrome (TCS), also known as mandibulofacial dysostosis and Franceschetti-Zwahlen- Klein syndrome, is an autosomal dominant disorder of soft tissue and the craniofacial bones. In most cases, TCS is the result of a mutation in the *TCOF1* gene. The incidence is estimated to be between 1/10,000 and 1/50,000 live births. Our purpose was to describe a case report of patient with TCS born in the Department of Neonatology at the Pomeranian Medical University in Szczecin (Poland) and his family with short review of literature.

**Case presentation:**

Clinical abnormalities which were found after birth mainly affect the head – hypoplasia of the cheek bones and the zygomatic bones, micrognation, deformed auricles with undeveloped external auditory canals, retrognathia of the mandible, cleft hard and soft palate and narrow palpebral fissure.

**Conclusions:**

The treatment of children with TCS is long-term. Patients require a series of reconstructive and plastic surgical procedures. Our patient presented the complete form of TCS. There are multiple surgeries awaiting him, which, eventually, will improve his quality of life.

## Introduction

Treacher Collins syndrome (TCS), also known as mandibulofacial dysostosis or Franceschetti-Zwahlen-Klein syndrome, is a developmental disorder of soft tissue and the craniofacial bones. It is a rare genetic disease inherited as an autosomal dominant manner. The incidence of this syndrome is estimated to be 1/10,000–1/50,000 live births. 

**Figure 1:**
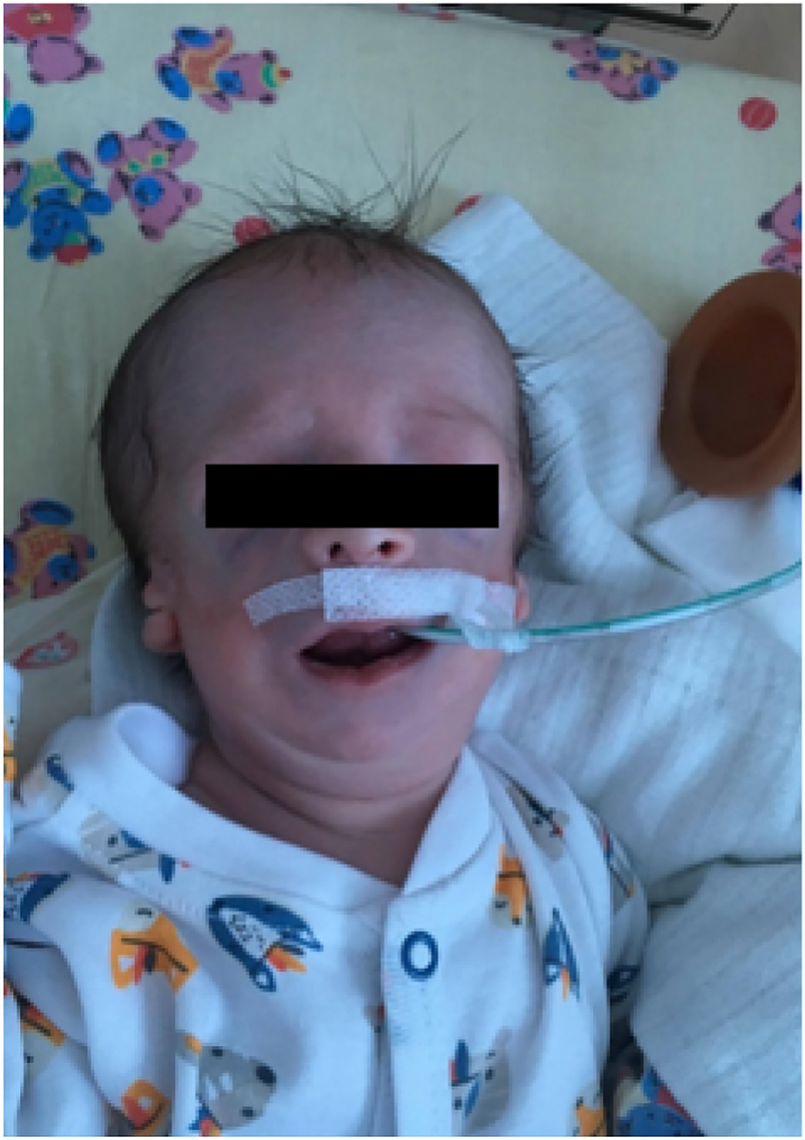
Our patient with Treacher Collins syndrome after birth.

**Figure 2:**
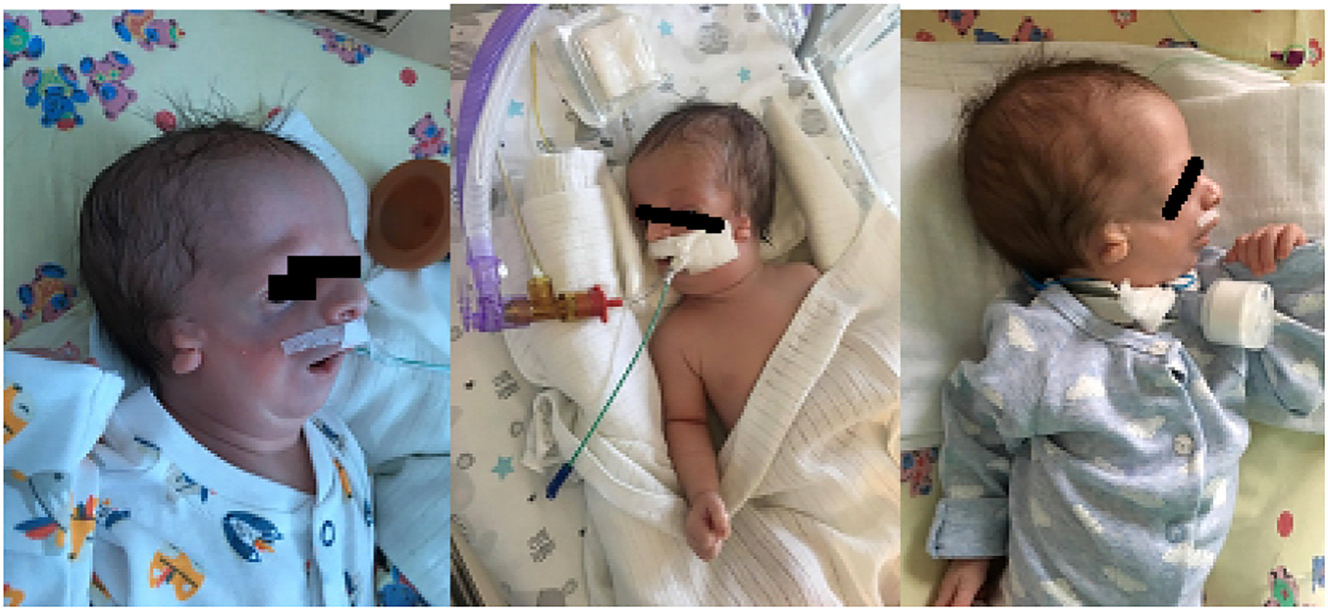
Typical features of Treacher Collins syndrome in terms of developmental craniofacial disorders in our patient.

**Figure 3:**
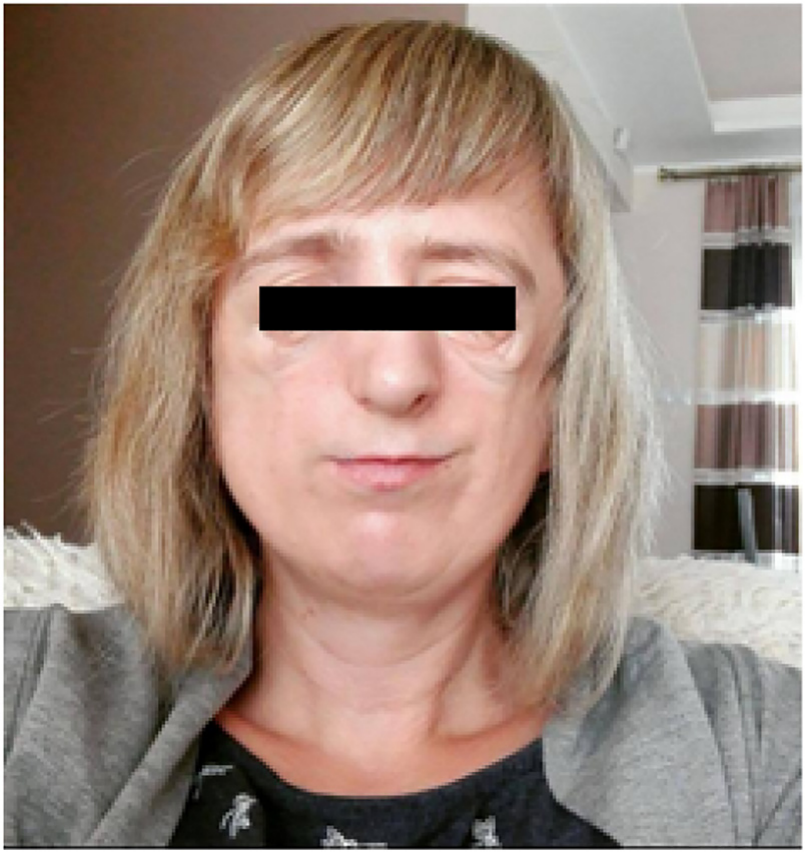
The mother of our patient with the features of Treacher Collins syndrome after surgeries.

In most cases, TCS is the result of a mutation in the *TCOF1* gene: 60% is a *de novo* mutation and 40% is familial. This disease is characterized by many abnormalities–mainly affecting the head and neck. As a result of the *TCOF1* gene mutation-the apoptosis of the neural crest cells increases and their proliferation decreases, which results in craniofacial disorders. The primary symptom is hypoplasia of the facial bones, mainly the mandible and the zygomatic bone. The mandible is poorly developed and the base of the eye socket is incomplete, while the palate is deformed, being high-arched, or “gothic”, although a cleft is rare [1], [2], [3].

Some patients have atrophy of the posterior nostrils, which results in respiratory distress after birth. The typical “bird-face” or “fish-face” results from bone lesions with hypoplasia of the lateral and medial pterygoid muscles. The features of TCS include slanting palpebral fissures. Coloboma in the lower eyelids, the absence of eyelashes and lower tear ducts are often observed. 77% of patients have hearing disorders [1]. These generally involve the external auditory canal and middle ear. The auriculars are deformed, while the external auditory canal is narrowed or overgrown. These disorders lead to hearing loss or total deafness. Anatomical or functional changes in the inner ear have not been observed. Most patients with TCS have dental abnormalities: paramolar teeth (Supernumerary Teeth), absence of tooth buds, enamel hypoplasia, or ectopic tooth position [1, 2, 4].

Diagnosis of TCS relies upon the characteristic appearance and identification of the mutated gene which is responsible for the occurrence of the symptoms. Ultrasound examination is helpful during pregnancy. If there is a family history of the condition, early diagnosis is possible in the first trimester of pregnancy, based on molecular tests.

## Case presentation

S.W. is a newborn of 2nd gestation, 2nd delivery, was born at full-term (40 Hbd) by vaginal delivery, in good general condition (Apgar 9/9/9) with a body weight of 2,940 g. He was born in the Department of Neonatology at the Pomeranian Medical University in Szczecin (Figure 1). 

During examination after birth, numerous abnormalities in the craniofacial structures were found: hypoplasia of the cheek bones, hypoplasia of the zygomatic bones, micrognation, deformed and residual auricles, undeveloped external auditory canals on both sides, retrognathia of the mandible, cleft hard and soft palate, narrow palpebral fissure. Despite the numerous developmental disorders, the neonate was initially able to breathe.

Respiratory failure was observed from the second day of life. On day 3 non-invasive ventilation (n-CPAP) was used, but with disappointing results. Retrognathic mandible and collapsing tongue, together with anxiety in the child, caused an increase in respiratory failure. Non-invasive ventilation was stopped and it was necessary to apply an oropharyngeal tube. These actions have failed to improve clinical condition.

There was a suddenly clinical deterioration on day 11 resulting in commencing non-invasive ventilation (initially SIMV mode Fi02 30%). The next day, patient suffered cardiac arrest. Because of increased inflammation laboratory parameters and inflammatory lesions in the chest X-ray, he was treated with a combined antibiotic therapy.

Infection was confirmed by a positive blood culture results. Despite the treatment and respiratory support by non-invasive ventilation, respiratory failure increased. On day 15th, patient was transferred to the Pomeranian Medical University SPSK1 Department of Intensive Care for Children in order for a tracheostomy to be performed (Figure 2).

During hospitalization, diagnostics was extended by a CT scan of the head, neck, chest, abdomen and pelvis. The study did not show any lesions in the brain, but there were craniofacial defects typical for TCS and pneumothorax, which required chest drain insertion.

On the 26th day of life, he was transferred back to our Department to continue treatment. The general condition of our patient was stable at admission, and antibiotic therapy was continued. The patient had to be aspirated frequently because of the large amount of mucous-white secretions in the tracheostomy tube.

In supporting therapy, we used inhalations and chest clapping with good results. The patient was fed through a naso-gastric tube because of the anatomical disorders in his mouth and no sucking reflex. Initially, modified milk for full-term newborns was used in nutrition, but because of poor weight gain, we introduced high protein diet with good effect.

During hospitalization, the infant was seen by an otolaryngologist, ophthalmologist, geneticist and surgeon. Genetic tests identified the p.Gln329Ter variant in one allele of the TCOF1* gene, which confirmed the clinical diagnosis of TCS.

Further diagnostics and treatment of the developmental facial defects are planned at the Craniofacial Defects Clinic after 3 months of age. The first correction of the cleft palate is planned when he reaches 1 year old.

Following all the diagnostics, the 2-month-old boy was discharged and went home under the care of a home hospice, having agreed the dates for the next consultations.

The mother of our patient also presented typical features of TCS in terms of developmental craniofacial disorders: hypoplasia of the auricles, external auditory canals and middle ear, no mastoid processes and hearing loss, hypoplasia of the jaw, branches of the jaw, cleft palate and lower eyelids. She needed multi-stage surgical treatment during her childhood (Figure 3).

At the age of 4, she had correction of her cleft soft palate, followed by reconstruction of both auricles with a cartilage graft from the rib arch, the left at age 10 and the right at age 11. The next stage was transplantation of bone into the right and left zygomatic area and plastic surgery on the eyelids of the left eye. The mother had not received a genetic diagnosis. Additionally, her 11-year-old daughter also has typical TCS features. The father does not present any clinical symptoms of TCS.

## Discussion

TCS is a rare genetic disease. The incidence is estimated to be between 1/10,000 and 1/50,000 live births [1, 5]. A group of people with undiagnosed TCS exists, because there in an incomplete form of this syndrome with low severity of developmental craniofacial disorders.

The first reports of this syndrome were published in 1846, when Thomson described three patients with craniofacial developmental disorders. The next description of a familial occurrence of mandibulofacial dysostosis appeared in 1899. In 1900, Treacher Collins described another 2 patients and Franceschetti and Klein defined the syndrome 49 years later. They standardized the nomenclature and classified the syndrome according to the intensity of the symptoms [1, 5].

The clinical symptoms of TCS are quite characteristic and they are the result of the mutation of 1 of 3 genes (*TCOF1*, *POLR1C* or *POLR1D*) on chromosome 5 [1, 3, 5–8].

In approximately 90% of cases, TCS is caused by mutations in the *TCOF1* gene, which encodes the treacle phosphoprotein. This plays an important role in the early developmental stage of facial tissues, which initially causes increased apoptosis of neural crest cells and then inhibits cranial development. The effects are hypoplasia of the zygomatic bones, cheekbones, mandible and palate, which can cause respiratory disorders as well as problems with nutrition. Developmental disorders of the eye bones result in the characteristic position of the eyes, while developmental disorders of the auditory canals cause hearing loss or deafness [1, 3, 5–9].

The disease is autosomal dominant inherited if affects the *TCOF1* or *POLRC1C* gene. In most of cases is the result of *de novo* mutation [3]. The symptoms and severity of TCS can be very significantly, even if there is a family history of it. Most often, the intellectual development of the patients is normal. Only 5% patients have neurological problems related to psychomotor developmental retardation.

We should remember that in TCS, also known as mandibulofacial dysostosis or Franceschetti-Zwahlen-Klein syndrome, developmental disorders are limited only to the craniofacial bones and soft tissues. This syndrome should be differentiated from other syndromes where there are limb anomalies in addition to the typical disorders present in TCS. For example, in Nager syndrome, there is additional hypoplasia or aplastic thumbs, aplasia in one of the forearm bones or fused ulna and radius bones. Some patients have camptodactyly and/or syndactyly.

Nager syndrome is caused by a mutation in the *SF3B4* gene located on the 1q12-q21 chromosome. In Miller syndrome, hypoplasia of the long bones, aplasia or incorrect position of the fingers, formation of synostosis, which shortens the length of the limbs, in addition to hypoplasia of the craniofacial bones all occur. A mutation in the *DHODH* gene located on the 16q22.2 chromosome causes Miller syndrome. Cincinnati type dysostosis should also be included in the differential diagnosis of TCS. The characteristic features are short and broad fingers, a short arched femur, and delayed or absent ossification [5].

In the case under discussion, we found no other bone changes besides craniofacial bone disorders. The family history of TCS in the patient’s mother and sister, with a positive genetic test, allows us to confirm the family occurrence of the mutations, which is much more interesting due to the lower prevalence of familial TCS.

There are different forms – from complete to atypical, depending on the severity of the clinical manifestations [10]. Our patient presented the complete form of this syndrome, with developmental anomalies including the eyes, ears, nose, lips and facial bones.

Mandibular hypoplasia and a collapsing tongue can cause respiratory disorders, which may lead to respiratory failure, often lead to intubation sometimes requiring a tracheostomy, as with our patient [1, 2].

The treatment of children with TCS is long-term and requires the involvement of many specialists: craniofacial surgeons, plastic surgeons, otolaryngologists, orthodontists, geneticists, speech therapists and psychologists.

Patients require a series of reconstructive and plastic surgical procedures, such as cleft palate repair, zygomatic bone reconstruction, and reconstruction of the auricles in the external auditory canal. Soft tissue plastic surgery and long-term orthodontic treatment are also very important in the treatment [1, 4, 7, 8, 10].

In our case, the patient and his family presented the complete form of TCS. In the future the boy will require many visits to highly specialized centers. There are multiple surgeries awaiting him. which, eventually, will improve his quality of life.
